# Serum cytokine levels are associated with tumor progression during FOLFIRINOX chemotherapy and overall survival in pancreatic cancer patients

**DOI:** 10.3389/fimmu.2022.898498

**Published:** 2022-08-25

**Authors:** Fleur van der Sijde, Willem A. Dik, Dana A. M. Mustafa, Eveline E. Vietsch, Marc G. Besselink, Reno Debets, Bas Groot Koerkamp, Brigitte C. M. Haberkorn, Marjolein Y. V. Homs, Quisette P. Janssen, Saskia A. C. Luelmo, Leonie J. M. Mekenkamp, Astrid A. M. Oostvogels, Marja A. W. Smits-te Nijenhuis, Johanna W. Wilmink, Casper H. J. van Eijck, the Dutch Pancreatic Cancer Group

**Affiliations:** ^1^ Erasmus MC Cancer Institute, Department of Surgery, University Medical Center Rotterdam, Rotterdam, Netherlands; ^2^ Laboratory of Medical Immunology, Department of Immunology, Erasmus MC, University Medical Center Rotterdam, Rotterdam, Netherlands; ^3^ Tumor Immuno-Pathology Laboratory, Department of Pathology, Erasmus MC, University Medical Center Rotterdam, Rotterdam, Netherlands; ^4^ Cancer Center Amsterdam, Department of Surgery, Amsterdam UMC, University of Amsterdam, Amsterdam, Netherlands; ^5^ Laboratory of Tumor Immunology, Department of Medical Oncology, Erasmus MC, University Medical Center Rotterdam, Rotterdam, Netherlands; ^6^ Department of Medical Oncology, Maasstad Hospital, Rotterdam, Netherlands; ^7^ Department of Medical Oncology, Erasmus MC, University Medical Center Rotterdam, Rotterdam, Netherlands; ^8^ Department of Medical Oncology, Leiden University Medical Center, Leiden, Netherlands; ^9^ Department of Medical Oncology, Medisch Spectrum Twente, Enschede, Netherlands; ^10^ Cancer Center Amsterdam, Department of Medical Oncology, Amsterdam UMC, University of Amsterdam, Amsterdam, Netherlands

**Keywords:** pancreatic cancer, biomarker, treatment response, cytokine, IL-1RA

## Abstract

**Background:**

Biomarkers predicting treatment response may be used to stratify patients with pancreatic ductal adenocarcinoma (PDAC) for available therapies. The aim of this study was to evaluate the association of circulating cytokines with FOLFIRINOX response and with overall survival (OS).

**Methods:**

Serum samples were collected before start and after the first cycle of FOLFIRINOX from patients with PDAC (*n*=83) of all disease stages. Overall, 34 circulating cytokines were analyzed with a multiplex immunoassay. In addition, changes in peripheral blood immune cell counts were determined by flow cytometry to correlate with differences in cytokine levels. Chemotherapy response was determined by CT scans with the RECIST 1.1 criteria, as disease control (*n*=64) or progressive disease (*n*=19) within eight cycles of FOLFIRINOX.

**Results:**

Patients with high serum IL-1RA concentrations after one cycle of chemotherapy were less likely to have tumor progression during FOLFIRINOX (OR 0.25, *P*=0.040). Increase of circulating IL-1RA concentrations correlated with increase of total, classical (CD14+CD16-), and non-classical monocytes (CD14-CD16+), and dendritic cells. In multivariable cox regression, including the variables chemotherapy response outcome and baseline CA19-9 level, serum concentrations of IL-7 (HR 2.14, *P*=0.010), IL-18 (HR 2.00, *P*=0.020), and MIP-1β (HR 0.51, *P*=0.025) after one cycle of FOLFIRINOX showed correlations with OS.

**Conclusions:**

Circulating IL-1RA, IL-7, IL-18, and MIP-1β concentrations are biomarkers associated with FOLFIRINOX response in PDAC patients, suggesting an important role for specific immune cells in chemotherapy response and PDAC progression. Cytokine-based treatment might improve patient outcome and should be evaluated in future studies.

## Introduction

FOLFIRINOX is a combined chemotherapy regimen, including fluorouracil, leucovorin, irinotecan and oxaliplatin. It is currently the standard first-line treatment for locally advanced (LAPC) and metastatic pancreatic ductal adenocarcinoma (PDAC). Although the survival of patients with PDAC has improved with the implementation of this chemotherapy combination, the overall prognosis remains poor. Patients with LAPC have a median overall survival (OS) of approximately two years (1), whereas metastatic disease patients a median OS of 11 months after FOLFIRINOX treatment (2). Meanwhile, 60-70% of these patients will experience FOLFIRINOX-induced toxicity (1–3), affecting their quality of life. Therefore, biomarkers with the ability to predict treatment response or with prognostic properties are urgently needed to personalize treatment and to avoid unnecessary toxicity (4).

Prognostic biomarkers are biomarkers that can be used to identify the likelihood of a clinical event, recurrence of disease or disease progression. Predictive biomarkers can be used to identify individuals that will experience a favorable or unfavorable effect from exposure to a medical product (e.g. chemotherapy) compared to patients without this biomarker (5). The predictive value of a biomarker needs to be confirmed in a study including at least two treatment groups, preferably a randomized controlled trial, to prove its treatment-specific effect and exclude prognostic effects (6). For this patient population it could mean that patients not responding to FOLFIRINOX might benefit from other types of chemotherapy, such as gemcitabine with nab-paclitaxel.

Cancer cells display pro-inflammatory properties, inducing a tumor-promoting environment (7). PDAC is thought to be an inflammation-driven cancer; the tumor microenvironment includes predominantly pro-inflammatory instead of tumor-suppressive immune cells, supporting cancer progression (8). Several systemic inflammation markers, such as the systemic immune-inflammation index (9), neutrophil-to-lymphocyte ratio (10), or Glasgow prognostic score (11) are of prognostic significance and alterations can be detected even prior to PDAC diagnosis (12, 13).

The stromal microenvironment of PDAC is a complex structure of extracellular matrix, fibroblasts, and inflammatory cells (14). These inflammatory cells produce a variety of growth factors, cytokines, and chemokines (14, 15). Cytokines are signaling molecules that play an important role in the interaction and function of cells. Cytokines are mainly produced by immune cells, but also normal epithelial cells, stromal cells, fibroblasts, and cancer cells can produce both pro-inflammatory and anti-inflammatory cytokines (10). High levels of circulating immunosuppressive, tumor-promoting cytokines, such as transforming growth factor-beta (TGF-β), interleukin (IL)- 1β, IL-6, IL-8, and tumor necrosis factor-alpha (TNF-α), and lower levels of tumor-suppressive cytokines, e.g. IL-11, IL-12, and interferon-gamma (IFN-γ), have been found to correlate with poor prognosis in PDAC patients (16–21). Circulating cytokine concentrations might reflect tumor aggressiveness and immune status associated with tumor progression (22). Whether circulating cytokine levels can also predict the response to FOLFIRINOX is yet unknown. We hypothesized that dysregulation of the immune system, demonstrated by an increase of pro-inflammatory and decrease of anti-inflammatory cytokines, is prone to FOLFIRINOX non-response.

In this study, we evaluated the levels of circulating cytokine concentrations before and after one cycle of FOLFIRINOX, and assessed differences between patients with and without progressive disease at chemotherapy response evaluation CT scans. In addition, peripheral blood immune cell subsets were measured and correlations with changes in cytokine concentrations were determined in order to affirm the origin of these cytokines. Also, the association with early tumor progression during FOLFIRINOX and prognostic value for OS of individual cytokine markers was assessed.

## Materials and methods

This article was written according to the Reporting recommendations for tumor marker prognostic studies (REMARK) guidelines (23).

### Study design

Patients were selected from the local pancreatic biobank at the Erasmus MC, Rotterdam (MEC-2015-085) and participated in two multicenter, prospective trials conducted in the Netherlands. Patients with resectable or borderline resectable PDAC participated in the randomized clinical trial PREOPANC-2 (Dutch trial register NL7094, MEC-2018-004) comparing neoadjuvant FOLFIRINOX chemotherapy to neoadjuvant gemcitabine-based chemoradiotherapy, followed by surgical resection of the primary tumor (24). Patients with LAPC or metastatic PDAC participated in the iKnowIT study (Dutch trial register NL7522, MEC-2018-087), a prospective cohort study investigating the predictive value of circulating biomarkers. All trials were approved by the ethics committees of all participating centers: Erasmus MC, University Medical Center (Rotterdam, the Netherlands), Amsterdam UMC (Amsterdam, the Netherlands), Maasstad Hospital (Rotterdam, the Netherlands), and Medisch Spectrum Twente (Enschede, the Netherlands), and conducted in accordance with the declaration of Helsinki.

### Patient selection

All patients had histologically confirmed PDAC and were treated with first-line FOLFIRINOX between February 2015 and October 2019. Patients with resectable, borderline resectable, or locally advanced disease were scheduled for eight cycles of FOLFIRINOX and patients with metastatic disease for maximum twelve cycles of FOLFIRINOX, according to the PREOPANC-2 study protocol (resectable or borderline resectable disease) or the current standard of care in the Netherlands (LAPC and metastatic disease). Exclusion criteria were: age <18 years, co-medication with other chemotherapeutics, and previous treatment with FOLFIRINOX chemotherapy. A CT scan was performed before start of treatment and after each fourth cycle of chemotherapy to evaluate treatment response, based on the Response Evaluation Criteria in Solid Tumours (RECIST) 1.1 criteria, as part of standard clinical practice. Differences in circulating cytokine levels were tested between patients with disease control patients, including those with stable disease, partial response or complete response to FOLFIRINOX, and progressive disease patients, if CT evaluation showed progression within eight cycles of FOLFIRINOX.

Patient characteristics, such as age, sex, stage of disease, laboratory results, CT scan evaluations, and follow-up data were retrieved from medical records by a medical doctor. Follow-up ended upon the death of the patient. Due to the explorative character of this study, no formal sample size calculation was performed.

### Sample collection

Peripheral venous blood samples were collected before the start of chemotherapy and before the start of the second cycle of FOLFIRINOX, approximately two weeks later. Blood was collected in 10 mL serum tubes (Becton Dickinson, Franklin Lakes, NJ, USA) and 10 mL EDTA tubes (Becton Dickinson, Franklin Lakes, NJ, USA). Serum tubes were centrifuged at room temperature for 10 minutes at 1000g. Serum was then divided in aliquots and stored at -80°C until further use. Freshly obtained whole blood from EDTA tubes was used within 24 hours to enumerate immune cell populations.

### Cytokine detection

All serum samples were first analyzed with the ProcartaPlex Cytokine & Chemokine Convenience 34-Plex Human Panel 1A immunoassay (Invitrogen, Carlsbad, CA, USA, for detail see **Supplementary Table 1**), and measured using the Luminex MAGPIX system (Luminex, Austin TX, USA). Only cytokines detected in at least 70% of samples were used for further analyses. The cytokines IL-1β, IL-1RA, IL-2, and IL-18 were also measured using (high sensitivity) immunoassays from a different supplier (R&D systems, Minneapolis, MN, USA, **Supplementary Table 1**). In addition, soluble IL-2 receptor (sIL-2R) was quantified with enzyme-linked immunosorbent assay (ELISA; Diaclone, Besançon, France) as a sensitive marker for T-lymphocyte activation and regulator of IL-2-dependent cell function (25, 26).

Serum samples were subjected to a maximum of three freeze-thaw cycles and thawed on ice prior to use. Supernatants were loaded on Luminex or ELISA plates at the recommended dilutions with standard protein controls, according to the manufacturer’s instructions. For Luminex assays, cytokines were quantified by the analysis of raw data using xPONENT 4.2 software (Luminex, Austin, TX, USA). For the ELISA assay, standard curves were constructed and used to quantify sIL-2R. Further technical details are shown in **Supplementary Table 1**.

### Immune cell enumeration

Flow cytometry was performed to quantify main granulocyte, monocyte, and lymphocyte subsets (**Supplementary Table 2**). Peripheral blood samples for immunophenotyping were available for 50/83 patients (60.2%). Whole blood was stained with monoclonal antibodies (MoAb) and after lysis of red blood cells analyzed by multi-color FCM on a BD 3-laser Celesta flow cytometer using FACSDiva 8.x software (Becton Dickinson, Franklin Lakes, NJ, USA). An overview of the monoclonal antibodies used is available in **Supplementary Table 3**. Absolute cell counts were determined using Flow-Count Fluorospheres (Beckman Coulter, Brea, CA, USA). The MoAb panel has been optimized, and compensated using Fluorescence minus one (FMO) controls (27). Data were gated and analyzed using FlowJo software (Tree Star, San Carlos, CA, USA). The gating strategy is presented in **Supplementary Figure 1**.

### Statistical analysis

Detection rates of cytokines were compared between disease control and progressive disease patients using Fisher’s exact tests, and with Chi-squared test for the comparison between the different stages of disease. Absolute circulating cytokine concentrations and immune cell counts, and the percentage increase of cytokine concentrations and immune cell numbers between disease control and progressive disease patients were compared with Mann-Whitney U tests. The comparison of cytokine concentrations between patients with resectable, LAPC, and metastatic disease was calculated with Kruskal-Wallis tests. Correlations between alterations in cytokine concentrations and immune cell numbers were determined with Pearson’s correlation coefficient.

Univariable and multivariable binary logistic regression was performed to analyze the predictive value of circulating cytokine levels for tumor progression during FOLFIRINOX chemotherapy, including patient characteristics with known association with treatment outcome: stage of disease and baseline CA19-9 levels. Cytokine levels were dichotomized based on the median concentration for each individual cytokine per time point of measurement.

Overall survival (OS) was calculated as the time between the start of FOLFIRINOX and death. The prognostic value of circulating cytokine levels was tested with univariable and multivariable Cox regression analysis, including known prognostic factors: age, stage of disease, chemotherapy response, and baseline CA19-9 levels. Differences in median OS were derived from Kaplan-Meier curves whereby groups of patients with cytokine levels below or above the median concentration were compared using log-rank tests.

Only two-sided tests were used and *P*-values <0.05 were considered statistically significant. Data were analyzed using SPSS Statistics for Windows (version 25.0; IBM, Armonk, NY, USA).

## Results

### Patient characteristics

In total, cytokine data obtained from 166 samples from 83 PDAC patients were available for analysis. Patient characteristics are presented in **Table 1**. The cohort consisted of patients from all disease stages: 34% was diagnosed with resectable or borderline resectable disease, 42% with LAPC, and 24% with metastatic disease. In this cohort, 19 (23%) patients showed progressive disease during FOLFIRINOX.

**Table 1:** Patient characteristics.

	All patients, *n*=83 (%)
**Age (years), median (IQR)**	64 (58-70)
**Sex, male**	47 (57)
**Stage of disease**	
**Resectable or borderline resectable** ** Locally advanced** ** Metastatic**	28 (34) 35 (42) 20 (24)
**Response^a^ to FOLFIRINOX**	
** Stable disease** ** Partial response** ** Progressive disease**	54 (65) 10 (12) 19 (23)
**Response^a^ to FOLFIRINOX, dichotomized**	
**Disease control** **Progressive disease**	64 (77) 19 (23)
Time point of CT evaluation progressive disease* (n=19)	
** After cycle 1** ** After cycle 2** ** After cycle 3** ** After cycle 4** ** After cycle 6** ** After cycle 8**	1 (5) 3 (16) 2 (11) 9 (47) 1 (5) 3 (16)
**Number of cycles of FOLFIRINOX received, median (IQR)**	8 (4-8)
**Baseline CA19-9 (kU/L), median (IQR)**	320 (60-1296)

CA19-9, carbohydrate antigen 19-9; IQR, interquartile range. ^a^According to the RECIST 1.1 criteria.

### Cytokine detection rates and treatment response outcome

**Supplementary Table 4** gives an overview of the 34 cytokines and chemokines measured using a multiplex panel, showing their detection rate, mean concentrations with standard deviation, and median concentrations with interquartile range. GM-CSF, GRO-α, IL-5, IL-12p70, IL-23, IL-31, and TNF-β were not detected in any of the samples. Eotaxin, IP-10, MIP-1β, RANTES, SDF-1α were detected in all serum samples. Comparisons of cytokine detection rates between patients with disease control and patients with progressive disease during FOLFIRINOX are presented in **Table 2**. Before the start of FOLFIRINOX, IL-1β (28% vs 5%) and IL-2 (18% vs 0%) were more often detected in samples from disease control patients then in samples from progressive disease patients (both *P*=0.059). After one cycle of FOLFIRINOX, IL-1RA was more often detected in patients with disease control (48%) compared to patients with progressive disease (21%, *P*=0.038).

**Table 2:** Comparison of detection rates of cytokines and chemokines between patients with disease control and patients with progressive disease during FOLFIRINOX, measured with a multiplex Luminex panel.

Before start of FOLFIRINOX			
Cytokine/chemokine	Detection rate disease control (%), *n*=64	Detection rate progressive disease (%), *n*=19	*P*
**IFN-γ**	4 (6)	1 (5)	1.000
**IL-1α**	12 (19)	4 (21)	1.000
**IL-1β**	18 (28)	1 (5)	0.059
**IL-1RA**	2 (3)	1 (5)	0.547
**IL-2**	12 (19)	0 (0)	0.059
**IL-6**	1 (2)	1 (5)	0.408
**IL-7**	45 (70)	14 (74)	1.000
**IL-10**	2 (3)	0 (0)	1.000
**IL-15**	6 (9)	0 (0)	0.328
**IL-17A**	13 (20)	1 (5)	0.172
**IL-18**	29 (45)	6 (32)	0.428
**IL-21**	6 (9)	1 (5)	1.000
**IL-22**	5 ()	0 (0)	0.584
**IL-27**	5 (78)	0 (0)	0.584
**MCP-1**	62 (97)	18 (95)	0.547
**MIP-1α**	18 (28)	5 (26)	1.000
**TNF-α**	10 (16)	0 (0)	0.107
**After 1 cycle of FOLFIRINOX**			
**Cytokine/chemokine**	**Detection rate disease control (%), *n*=64**	**Detection rate progressive disease (%), *n*=19**	***P***
**IFN-γ**	6 (9)	1 (5)	1.000
**IL-1α**	11 (17)	3 (16)	1.000
**IL-1β**	14 (21)	4 (21)	1.000
**IL-1RA**	31 (48)	4 (21)	0.038^a^
**IL-2**	10 (16)	0 (0)	0.107
**IL-6**	4 (6)	2 (11)	0.616
**IL-7**	45 (70)	13 (68)	1.000
**IL-10**	3 (5)	0 (0)	1.000
**IL-15**	4 (6)	0 (0)	0.569
**IL-17A**	14 (22)	2 (11)	0.340
**IL-18**	44 (69)	11 (58)	0.416
**IL-21**	4 (6)	2 (11)	0.616
**IL-22**	4 (6)	1 (5)	1.000
**IL-27**	5 (8)	0 (0)	0.584
**MCP-1**	62 (97)	17 (90)	0.223
**MIP-1α**	13 (20)	4 (21)	1.000
**TNF-α**	8 (13)	1 (5)	0.677

IFN, interferon; IL, interleukin; IL-1RA, interleukin-1 receptor antagonist; IP-10, interferon gamma-induced protein 10; MCP, monocyte chemoattractant protein; MIP, macrophage inflammatory protein; TNF, tumor necrosis factor. ^a^Significant P-value. *P*-values are calculated by Fisher’s exact tests.

To improve the detection rates of IL-1β, IL-1RA, IL-2, and IL-18, these cytokines were re-analyzed with high sensitivity singleplex or duplex immunoassay from a different manufacturer (**Supplementary Table 1**). Eotaxin, IL-7, IP-10, MCP-1, MIP-1β, RANTES, and SDF-1α all met the inclusion criteria of detection in >70% of samples and were selected for further analyses. Together with the sIL-2R, a total of eleven cytokines were used to create predictive and prognostic models.

### Cytokine concentrations and treatment response outcome

In **Supplementary Table 5** we show the comparisons between cytokine detection rates and cytokine concentrations between patients with resectable, LAPC, and metastatic disease both before and after one cycle of FOLFIRINOX. There were no statistically significant differences between the different stages of disease and we did not perform additional analyses for the individual disease stage groups, also because of the limited number of patients per disease stage.

IL-1β and IL-2 results were only available for 88 samples (*n*=44 patients). The other samples could not be used due to absence of detectable bead signals. With individual assays, IL-1β, IL-1RA, IL-18 and sIL-2R were detected in all available samples. IL-2 was detected in only 28% of samples and results of this cytokine will therefore not be further discussed.

**Figure 1:**
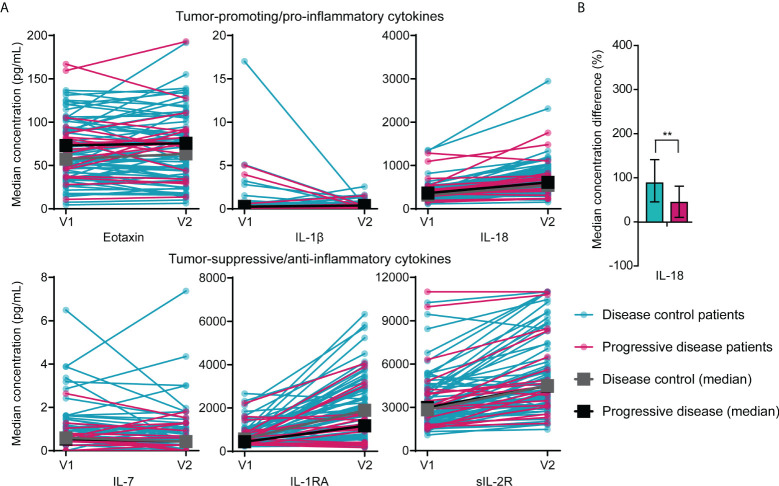
Differences in circulating cytokine concentrations between patients with disease control and patients with progressive disease during FOLFIRINOX treatment. **(A)** Concentrations of pro-inflammatory (eotaxin, IL-1β, and IL-18) and anti-inflammatory cytokines (IL-7, IL-1RA, sIL-2R) in serum of individual patients with disease control (*n*=64) and patients with progressive disease (*n*=19) before start of FOLFIRINOX (V1) and after one cycle of FOLFIRINOX (V2) and the median concentration over time for the two response groups. Median concentrations before or after one cycle did not show statistical significant differences between the two groups. **(B)** Percentage increase of serum IL-18 concentrations after one cycle of FOLFIRINOX in patients with disease control and progressive disease. **P < 0.01, calculated with Mann-Whitney U test.

There were no significant differences in median concentrations of any of the eleven cytokines (Eotaxin, IL-1β, IL-1RA, sIL-2R, IL-7, IL-18, IP-10, MCP-1, MIP-1β, RANTES, SDF-1α) between disease control and progressive disease patients in samples drawn before the start or after one cycle of FOLFIRINOX. However, pro-inflammatory/tumor-promoting cytokines (eotaxin, IL-1β, IL-18) seemed to be found in higher concentrations in progressive disease patients, while disease control patients showed higher levels of anti-inflammatory/tumor-suppressive cytokines (IL-1RA, sIL-2R, IL-7), as presented in **Figure 1A**.

In patients with disease control, IL-18 showed a larger increase during FOLFIRINOX (median increase 89%; IQR 46-142%) compared to patients with progressive disease (median increase 44%; IQR 11-81%), *P*=0.007), visualized in **Figure 1B**. An overview of cytokine concentrations during treatment for all cytokines investigated is included in **Supplementary Figure 2**.

### Cytokine concentrations in a predictive model

To investigate the value of individual cytokine concentrations to predict early tumor progression during FOLFIRINOX, a binary logistic regression model was created. Early tumor progression was defined as progression of disease during or immediately after FOLFIRINOX treatment, established on CT scans using the RECIST 1.1 criteria. From univariable analyses, the variables IL-1RA concentration after one cycle of FOLFIRINOX (OR 0.19, 95% CI 0.06-0.63), IL-18 before the start of FOLFIRINOX (OR 2.62, 95% CI 0.88-7.74), an increase of IL-18 (OR 0.33, 95% CI 0.11-0.94), and an increase of MIP-1β (OR 0.37, 95% CI 0.13-1.08) were selected for multivariable analyses. In this patient cohort, stage of disease and baseline CA19-9 level did not predict early tumor progression. The results of the univariable and multivariable analyses are presented in **Table 3**. In multivariable analysis, only IL-1RA concentration after one cycle of FOLFIRINOX remained an independent predictor of FOLFIRINOX response (OR 0.25; 95% CI 0.07-0.094, *P*=0.040). Patients with IL-1RA concentrations above the median of the measurements showed a lower risk of early tumor progression during FOLFIRINOX.

**Table 3:** Univariable and multivariable binary logistic regression model for the prediction of early tumor progression during FOLFIRINOX.

Variable	Univariable		Multivariable	
	OR for progressive disease(95% CI)	*P*	OR for progressive disease(95% CI)	*P*
**IL-1RA after 1 cycle of FOLFIRINOX**				
** <median** ** >median**	Ref 0.19 (0.06-0.63)	0.007^a^	Ref 0.25 (0.07-0.94)	0.040^a^
**IL-18 before start of FOLFIRINOX**				
** <median** ** >median**	Ref 2.62 (0.88-7.74)	0.083	Ref 2.08 (0.63-6.92)	0.231
**IL-18 increase during 1 cycle of FOLFIRINOX**				
** ≤50%** ** >50%**	Ref 0.33 (0.11-0.94)	0.038^a^	Ref 0.66 (0.119-2.27)	0.506
**MIP-1β increase during 1 cycle of FOLFIRINOX**				
** No** ** Yes**	Ref 0.37 (0.13-1.08)	0.069	Ref 0.73 (0.21-2.48)	0.729

CI, confidence interval; IL, interleukin; IL-1RA, interleukin-1 receptor antagonist; MIP, macrophage inflammatory protein; OR, odds ratio; Ref, reference. ^a^Significant P-value.

### Correlation between cytokines and immune cells

**Figure 2:**
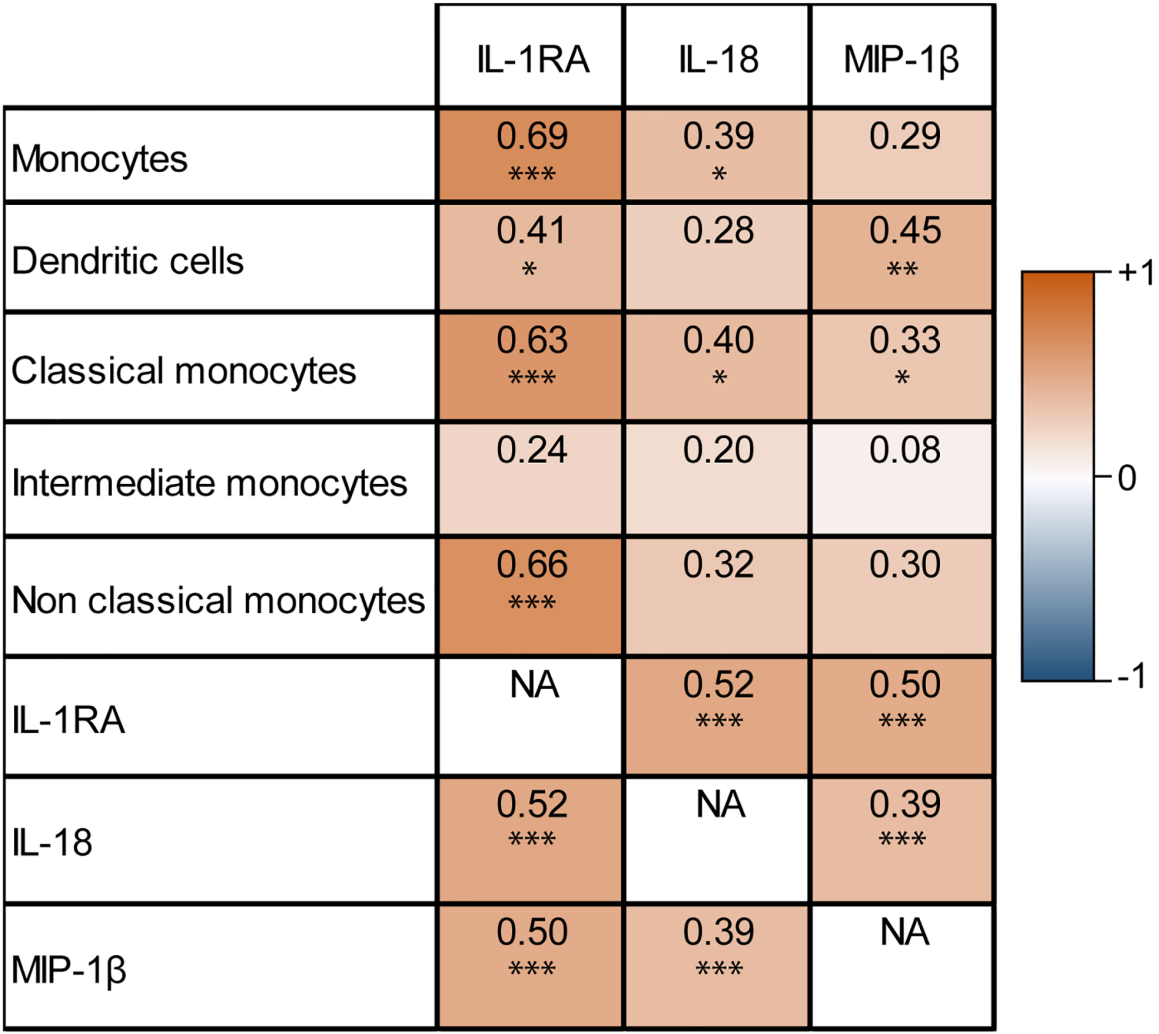
Correlation matrix of monocyte-related serum cytokine concentration increase and circulating monocyte cell number increase after one cycle of FOLFIRINOX. Increasing IL-1RA, IL-18, and MIP-1β concentrations after one cycle of FOLFIRINOX showed significant correlations with the increase of several subsets of monocytes. *P < 0.05, **P < 0.01, ***P < 0.001, calculated with Pearson’s correlation. NA = 'not applicable' in the correlation matrix.

IL-1RA, IL-18, and MIP-1β, predictors of early tumor progression in univariable analysis, are cytokines produced by monocytes (28–30). Therefore a correlation matrix was made between the percentage increase of serum concentrations of these cytokines and the percentage increase of monocyte cell numbers, based on results available from 50 patients (**Figure 2**). The increase of circulating IL-1RA concentrations correlated significantly with total monocytes (Pearson’s r=0.69, *P*<0.001), classical monocytes (r=0.63, *P*<0.001), non-classical monocytes, and dendritic cell (r=0.41, *P*=0.011) increase in the peripheral blood (r=0.66, *P*<0.001). IL-18 correlated with total monocytes (r=0.39, *P*=0.015), and classical monocytes (r=0.40, *P*=0.014). MIP-1β correlated with dendritic cells (r=0.45, *P*=0.004), and classical monocytes (r=0.33, *P*=0.046). Also, the cytokines in this correlation plot were correlated to the other cytokines as well (Pearson’s correlation between IL-1RA - IL-18 r=0.52, IL-1RA - MIP-1β r=0.50, IL-18 - MIP-1β r=0.39, all P<0.001), meaning that an increase after FOLFIRINOX for one cytokine is accompanied by the increase of the other cytokine concentrations.

**Figure 3:**
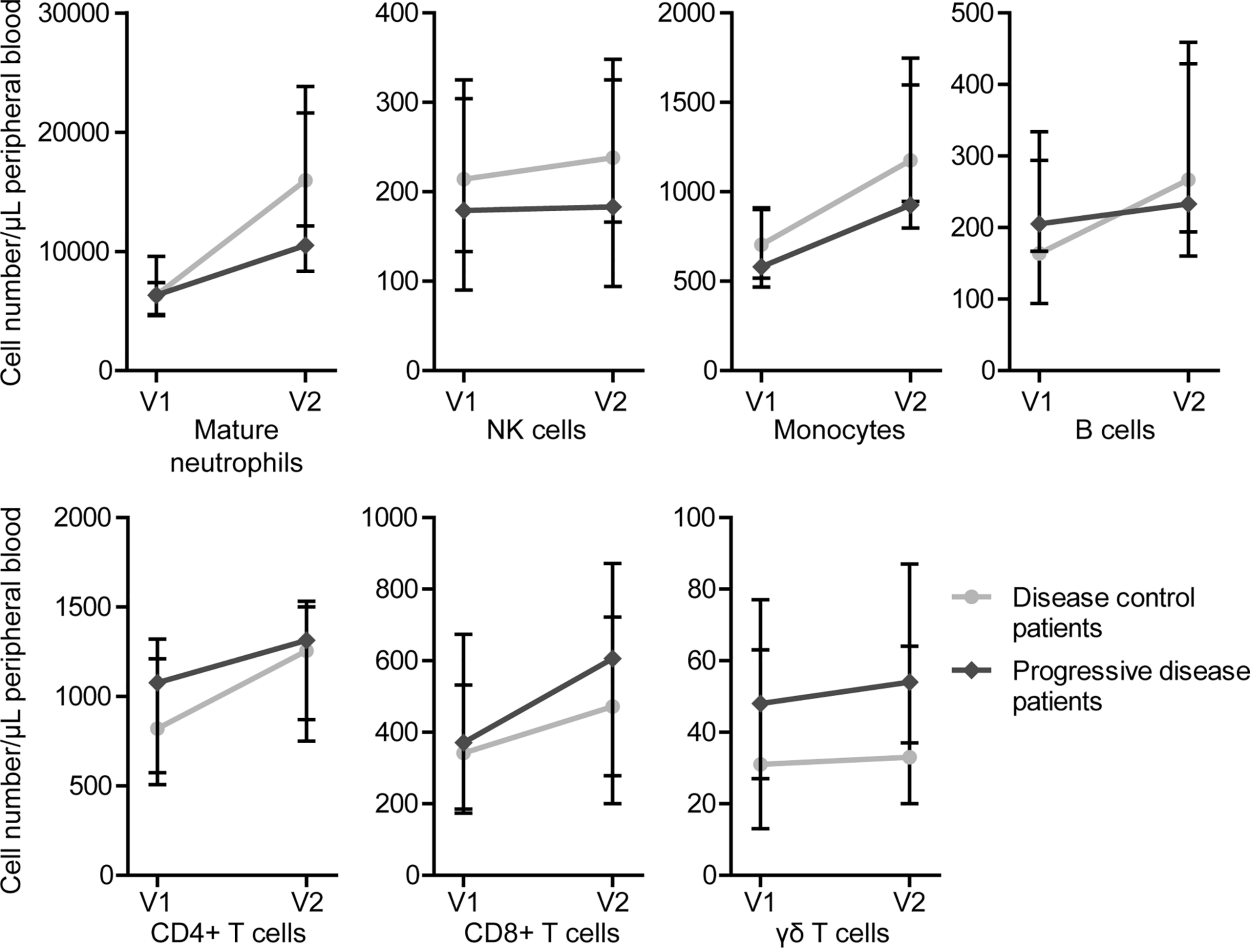
Circulating immune cell numbers in patients with disease control and patients with progressive disease before start of FOLFIRINOX (V1) and after one cycle of FOLFIRINOX (V2). Circulating cell numbers of mature neutrophils, NK cells, monocytes, B cells, and CD4+ T cells showed a larger increase in patients with disease control, CD8+ and γδ T cells in patients with progressive disease. Data is presented as median cell numbers with interquartile ranges.

Overall, an increase in all determined immune cell types was observed after one cycle of FOLFIRINOX (**Supplementary Figure 3**). There were no statistically significant differences in cell numbers before the start of FOLFIRINOX, after one cycle of FOLFIRINOX or in increase over time between disease control and progressive patients. However, we could detect a trend towards a stronger increase of tumor-suppressive cells (e.g. neutrophils, B cells, NK cells, and monocytes) in disease control patients, together with a slight increase of immunosuppressive cells (e.g. γδ T cells) in progressive disease patients, as shown in **Figure 3**. However, there was also an increasing trend in T cells visible, including T helper cells and/or regulatory T cells (CD4+) and cytotoxic T cells (CD8+), in progressive disease patients.

### Cytokines associated with overall survival

The median follow-up time was 16.5 months for patients alive at last follow-up. Median OS for the total cohort was 12.5 months. Median OS was different for patients of the three disease stages; resectable disease 13.2 months, LAPC 15.7 months, and metastatic disease 9.0 months (*P*=0.008). In univariable analyses, IL-1RA concentration after FOLFIRINOX (HR 0.64 for concentrations above the median of measurements), sIL-2R before the start of FOLFIRINOX (HR 1.55), IL-7 after FOLFIRINOX (HR 1.57), IL-18 before (HR 1.56) and after FOLFIRINOX (HR 1.57), and MIP-1β after FOLFIRINOX (HR 0.63) were statistically significant predictors for OS, as shown in **Supplementary Table 6**. In multivariable analysis, including the variables baseline CA19-9 and RECIST treatment response outcome, IL-7 (HR 2.14; 95% CI 1.20-3.80, *P*=0.010), IL-18 (HR 2.00; 95% CI 1.11-3.60, *P*=0.020), and MIP-1β (HR 0.51; 95% CI 0.28-0.92, *P*=0.025) concentrations measured after one cycle of FOLFIRINOX remained significant prognostic factors for OS after FOLFIRINOX treatment. Patients with a high level of IL-7 and IL-18, and low level of MIP-1β were at risk for shorter OS. Stage of disease was not a prognostic variable in our Cox proportional hazards model (LAPC HR 0.61, *P*=0.113, metastatic disease HR 1.63, *P*=0.139). In **Supplementary Figure 4** Kaplan-Meier curves are shown for overall survival in patients with cytokine levels below or above the median cytokine concentration in this cohort for IL-1RA, IL-7, IL-18, and MIP-1β. High IL-1RA and MIP-1β and low IL-7 concentrations after one cycle of FOLFIRINOX show a trend of better overall survival after FOLFIRINOX treatment, though not statistically significant (respectively *P*=0.095, *P*=0.074, and *P*=0.087). IL-18 levels after FOLFIRINOX do not influence overall survival in these Kaplan-Meier curves (*P*=0.789).

## Discussion

In this multicenter study, we investigated serum cytokine concentrations in PDAC patients treated with FOLFIRINOX and the correlations of these cytokines with treatment response and prognosis. We found that most cytokines showed increasing concentrations after one cycle of FOLFIRINOX compared to baseline. However, IL-18 showed a stronger increase in patients responding to treatment. Low IL-1RA serum concentrations after one cycle of FOLFIRINOX were associated with an increased risk of early tumor progression during FOLFIRINOX. In addition, high IL-18 and IL-7, and low MIP-1β concentrations after one cycle of FOLFIRINOX were poor prognostic factors for OS. Our results support the hypothesis that prognosis in PDAC patients is at least partially determined by the activation level of the immune system and its response to cancer cells (7). We found a clear serum cytokine pattern in patients with disease control and patients with progressive disease during FOLFIRINOX. Favorable, anti-inflammatory cytokines, such as IL-1RA, IL-7, and MIP-1β were detected in higher concentrations in responding patients, while pro-inflammatory, tumor-promoting cytokines, such as IL-18 and IL-1β, were higher in patients with early progressive disease.

The correlation we observed between IL-1RA, IL-18 and MIP-1β with different monocyte subsets might indicate that differences in cytokine concentrations are related to differences in the immune environment and cellular response to chemotherapy. Though, the strong increase of T cells, including cytotoxic T cells (CD8+) and CD4+ cells including both T helper cells as well as regulatory T cells, in progressive disease patients, was unexpected. However, it is not clear whether the increase of all the investigated cell types might be explained by the increased cell proliferation in reaction to chemotherapy-induced cell death, by the administration of granulocyte colony-stimulating factor (G-CSF) after every chemotherapy cycle, or as a result of the proliferative effect on immune cells of FOLFIRINOX (31). Furthermore, we did not investigate the functional status and exhaustion rate of CD4+ and CD8+ cell populations, which is crucial in further studies.

The results found in this study are affirmed by existing literature on circulating cytokine levels and outcome in PDAC patients. Circulating IL-18 levels were previously found to increase during treatment with gemcitabine in combination with 5-FU or oxaliplatin, and higher IL-18 levels were associated with shorter OS (32). IL-1β is thought to facilitate tumor growth, angiogenesis, and metastasis in PDAC and may therefore negatively influence patient prognosis (17, 19). Both IL-18 and IL-1β are associated with objective gemcitabine-based chemotherapy response (19, 33). Also, MIP-1β and RANTES have been shown to associate with PDAC patient outcome (34, 35).

An interesting finding is that most cytokines with prognostic value in our study relate to the nuclear factor-κB (NF-κB) pathway, which plays a crucial role in the regulation of a plethora of inflammatory genes (36). The NF-κB signaling cascade is usually activated by pathogens, damaged tissue, and necrotic cells, and it includes a negative feedback loop, regulating its own activity (37). In cancer environments the NF-κB signaling pathway is continuously activated, resulting in constant production of large amounts of pro-inflammatory cytokines. Activation of the NF-κB pathway promotes all hallmarks of cancer: tumor cell proliferation and survival, angiogenesis, metastasis, and immune suppression (36, 38). IL-1β, which was found in higher concentrations in patients with poor prognosis, is a known activator of the NF-κB pathway (39). Also, several DNA mutations can activate the pathway (39, 40), including *KRAS* and *TP53* mutations which are found in almost all PDAC tumors (41). IL-1RA, the natural antagonist of IL-1, may regulate activation of the NF-κB pathway, thus reducing the negative effects of the pathway and improving patient prognosis (40).

Other cytokines which are also controlled by NF-κB were not or only in a limited number of patient samples detected. For example, TNF-α, IL-6, and IL-8, cytokines that have been shown to be upregulated in PDAC patients (17, 19, 42), were only detected in a limited number of samples. The relatively low serum cytokine concentrations and therefore lack of detection, is probably the most important limitation of this study. The cytokine concentrations measured in our study seem to be low in comparison to other studies with PDAC patients (17, 19, 35). This may be related to the immunoassay used to detect the cytokines. We chose to start our pilot project with a broad multiplex panel to screen for 34 different cytokines. Only small serum volumes were needed, which was an advantage because of the limited availability of serum from our patients, included in large multicenter, prospective studies. However, the small sample volumes in the discovery panel might explain the low detection rate of cytokines and increased sensitivity with the individual cytokine assays using larger serum volumes.

In this pilot study, the sample size was relatively small and cytokine concentrations and immune cell numbers varied widely between patients, also resulting in wide interquartile ranges for the median IL-1RA concentration. Most of our findings did therefore not reach statistical significance and we could only highlight some trends in cytokine and cell count differences between treatment response groups. The number of variables analyzed in this study might have led to a type I error. Though, we deliberately did not correct our raw data for multiple testing since this could result in type II errors, which are acceptable in exploratory studies such as this one. Unfortunately, subgroup analyses for the individual disease stages could not be performed due to the limited number of patients. Though, we did not find differences in cytokine detection rates or concentrations between patients of different disease stages.

In this study, we focus on responders and non-responders to FOLFIRINOX. In our dataset, the objective response rate (ORR) was 12% and the disease control rate 77%. Especially the ORR is lower than reported outcomes from other FOLFIRINOX clinical trials with ORR varying between 16-40% for metastatic patients (3, 43) and 17-30% for LAPC patients (41, 44, 45). The benefit of treatment, however, is often overestimated due to exclusion of patients with poor prognosis in clinical trials and are therefore not comparable to real-world data.

In the future, the predictive and prognostic value of IL-1RA must be confirmed in a large clinical trial including two different treatment arms, preferably a randomized clinical trial comparing FOLFIRINOX to gemcitabine with nab-paclitaxel outcomes. The future clinical implications of this study should include the evaluation of the additive effects of cytokine-based therapy on chemotherapy response and survival in PDAC patients. Studies with mouse models have already shown promising results of treatment with anakinra, an FDA-approved human IL-1RA protein drug used for rheumatoid arthritis. Anakinra inhibits IL-1 and by that the NF-κB pathway, reducing proliferation, migration, and invasion of PDAC cells, and IL-1 neutralization sensitizes cancer cells for immunotherapy and chemotherapy (40, 46). At this moment, two clinical trials are investigating the benefit of anakinra in addition to FOLFIRINOX (ClinicalTrials.gov identifier: NCT02021422) or gemcitabine chemotherapy (NCT02550327) in PDAC patients. Restoring the imbalance of tumor-promoting and tumor-suppressing components of the immune system might be the future of PDAC treatment.

## Conclusion

Low circulating IL-1RA cytokine concentrations are associated with an increased risk of early tumor progression during FOLFIRINOX. High IL-18 and IL-7, and low MIP-1β levels are poor prognostic factors for OS in PDAC patients. This indicates that activation and changes in the systemic immune response might play an important role in chemotherapy response and PDAC progression. Cytokine-based treatment might improve patient outcome and should be evaluated in future studies.

## Data availability statement

The raw data supporting the conclusions of this article will be made available by the authors, without undue reservation.

## Ethics statement

The studies involving human participants were reviewed and approved by the Ethics Committee of Erasmus MC, University Medical Center Rotterdam. The patients/participants provided their written informed consent to participate in this study.

## Author contributions

CE, EV, and WD supervised the project. FS, CE, DM, MB, BK, BH, MH, QJ, SL, LM, and JW provided the resources for the project. FS, MS-t, AO, and RD were responsible for the investigation. FS analyzed and visualized the data and wrote the original draft. All other authors reviewed and edited the manuscript before submission.

## Funding

This research was funded in part by the Support Casper foundation and by the Eurostars project (project number ESTAR17104).

## Acknowledgments

The authors would like to thank all patients for donating blood and participating in our study. Moreover, we thank J. Dumas for processing and storage of the blood samples, and M. Moskie, S. Snapper, E. Pijnappel, A. Stam, and J. Hans-Adema for their help with the collection of patient samples.

## Conflict of interest

The authors declare that the research was conducted in the absence of any commercial or financial relationships that could be construed as a potential conflict of interest.

## Publisher’s note

All claims expressed in this article are solely those of the authors and do not necessarily represent those of their affiliated organizations, or those of the publisher, the editors and the reviewers. Any product that may be evaluated in this article, or claim that may be made by its manufacturer, is not guaranteed or endorsed by the publisher.
