# Supplementary material for: Serum cytokine levels are associated with tumor progression during FOLFIRINOX chemotherapy and overall survival in pancreatic cancer patients
Source: Front Immunol. 2022 Aug 25;13:898498. doi: 10.3389/fimmu.2022.898498 (PMC9454314; doi:10.3389/fimmu.2022.898498)
Supplement: Supplementary file 1 [file DataSheet_1.docx]

Supplementary Material

**Supplementary Table 1.** Technical details of the used immunoassays.

| **Cytokine/chemokine** | **Immunoassay** | **Company** | **Amount of serum used** | **Dilution** |
| --- | --- | --- | --- | --- |
| Eotaxin, GM-CSF, GRO-α, IFN-α, IFN-γ, IL-1α, IL-1β, IL-1RA, IL-2, IL-4, IL-5, IL-6, IL-7, IL-8, IL-9, IL-10, IL-12p70, IL-13, IL-15, IL-17A, IL-18, IL-21, IL-22, IL-23, IL-27, IL-31, IP-10, MCP-1, MIP-1α, MIP-1β, RANTES, SDF-1α, TNF-α, TNF-β | Luminex ProcartaPlex [Cytokine & Chemokine Convenience 34-Plex Human Panel 1A](https://www.thermofisher.com/order/catalog/product/EPXR340-12167-901) | Invitrogen | 12.5 µL | 1:2 |
| IL-1β, IL-2 | Luminex Performance Human High Sensitivity Cytokine Magnetic Panel B | R&D systems | 25 µL | 1:2 |
| IL-1RA | Luminex Performance Human XL Cytokine Discovery Magnetic Panel | R&D systems | 17.5 µL | 1:2 |
| IL-18 | Luminex Human Magnetic Assay | R&D systems | 25 µL | 1:2 |
| sIL-2R | ELISA | Diaclone | 20 µL | 1:5 |

GM-CSF = granulocyte-macrophage colony-stimulating factor, GRO = growth-regulated oncogene, IFN = interferon, IL = interleukin, IL-1RA = interleukin-1 receptor antagonist, IP = interferon γ-induced protein, MCP = monocyte chemoattractant protein, MIP = macrophage inflammatory protein, RANTES = regulated upon activation, normal T cell expressed and secreted, SDF = stromal cell-derived factor, sIL2R = soluble interleukin-2 receptor, TNF = tumor necrosis factor.

**Supplementary Table 2.** Flow cytometry markers used for immunophenotyping of whole blood.

|  | **Markers** |
| --- | --- |
| **Granulocytes** | |
| Eosinophils | CD45^+^ SSC  CD15^+^  CD16^-^ |
| Mature neutrophils | CD45^+^ SSC  CD15^high^  CD16^high^ |
| Immature neutrophils | CD45^+^ SSC  CD15^+^  CD16^+^ |
| **Monocytes** | |
| Classical monocytes | CD45^+^ SSC  CD14^+^  CD16^-^ |
| Intermediate monocytes | CD45^+^ SSC  CD14^+^  CD16^+^ |
| Non-classical monocytes | CD45^+^ SSC  CD14^-^  CD16^+^ |
| Dendritic cells | CD45^+^ SSC  CD14^-^  CD16^-^  CD11c^+^ |
| Myeloid-derived suppressor cells (MDSCs) | CD45+ SSC  CD14+  CD16-  CD11b+  HLA-DR^low^ |
| **Lymphocytes** | |
| B cells | CD45^+^ SSC  CD3^-^  CD19^+^ |
| NK cells | CD45^+^ SSC  CD3^-^  CD56^+^  CD16^+/-^ |
| T cells | CD45^+^ SSC  CD3^+^ |
| αβ T cells | CD45^+^ SSC  CD3^+^  TCRαβ^+^  CD4^+^/CD8^+^ |
| γδ T cells | CD45^+^ SSC  CD3^+^  TCRγδ^+^ |

CD = cluster of differentiation, NK = natural killer, SSC = side scatter, TCR = T cell receptor.

**Supplementary Table 3.** Monoclonal antibodies used for flow cytometry analysis of whole blood.

| **Antibody** | **Clone** | **Supplier** | **Dilution** |
| --- | --- | --- | --- |
| CD3-PB | UCHT1 | BD Biosciences | 1:50 |
| CD4-V500 | RPA-T4 | BD Biosciences | 1:50 |
| CD8-APC-Cy7 | SK1 | BD Biosciences | 1:200 |
| CD11b-APC | D12 | BD Biosciences | 1:40 |
| CD11c-APC-eF780 | BU15 | eBioscience | 1:50 |
| CD14-FITC | M φP9 | BD Biosciences | 1:20 |
| CD15-PE | HI98 | BD Biosciences | 1:20 |
| CD16-PE-Cy7 | 3G8 | BD Biosciences | 1:400 |
| CD19-V500 | HIB19 | BD Biosciences | 1:50 |
| CD45-PerCP | 2D1 | BD Biosciences | 1:50 |
| CD56-APC | TULY56 | eBioscience | 1:40 |
| HLA-DR-BV786 | G46-6 | BD Biosciences | 1:80 |
| TCRgd-FITC | 11F2 | BD Biosciences | 1:20 |

**Supplementary Table 4.** Overview of serum cytokine detection rates and concentrations of the 34 cytokines and chemokines measured with the ProcartaPlex multiplex immunoassay.

|  | **Before start of FOLFIRINOX (*n*=83)** | | | **After 1 cycle of FOLFIRINOX (*n*=83)** | | |
| --- | --- | --- | --- | --- | --- | --- |
| **Cytokine/**  **chemokine** | **Detection rate (%)** | **Mean concentration, pg/mL (±SD)** | **Median concentration, pg/mL (IQR)** | **Detection rate (%)** | **Mean concentration, pg/mL (±SD)** | **Median concentration, pg/mL (IQR)** |
| **Eotaxin/CCL11** | 83 (100) | 67.28 (±36.24) | 61.11 (36.06-90.96) | 83 (100) | 71.92 (±40.93) | 72.12 (37.23-94.49) |
| **GM-CSF/CSF2** | 0 (0) | NA | NA | 0 (0) | NA | NA |
| **GRO-α/CXCL1** | 0 (0) | NA | NA | 0 (0) | NA | 0.00 (0.00-0.00) |
| **IFN-α** | 2 (2) | 0.09 (±0.72) | 0.00 (0.00-0.00) | 0 (0) | NA | NA |
| **IFN-γ** | 5 (6) | 0.15 (±0.70) | 0.00 (0.00-0.00) | 7 (8) | 0.14 (±0.55) | 0.00 (0.00-0.00) |
| **IL-1α** | 16 (19) | 9.14 (±48.28) | 0.00 (0.00-0.00) | 14 (17) | 8.98 (±48.82) | 0.00 (0.00-0.00) |
| **IL-1β** | 19 (23) | 0.82 (±3.98) | 0.00 (0.00-0.00) | 18 (22) | 0.57 (±1.99) | 0.00 (0.00-0.00) |
| **IL-1RA** | 3 (4) | 12.79 (±82.01) | 0.00 (0.00-0.00) | 35 (42) | 623.14 (±1279.31) | 0.00 (0.00-1063.19) |
| **IL-2** | 12 (15) | 1.22 (±5.50) | 0.00 (0.00-0.00) | 10 (12) | 0.47 (±1.47) | 0.00 (0.00-0.00) |
| **IL-4** | 1 (1) | NA | NA | 0 (0) | NA | NA |
| **IL-5** | 0 (0) | NA | NA | 0 (0) | NA | NA |
| **IL-6** | 2 (2) | 0.03 (±0.19) | 0.00 (0.00-0.00) | 6 (7) | 0.25 (±1.29) | 0.00 (0.00-0.00) |
| **IL-7** | 59 (71) | 0.85 (±1.09) | 0.54 (0.00-1.11) | 58 (70) | 0.71 (±1.07) | 0.42 (0.00-0.93) |
| **IL-8** | 0 (0) | NA | NA | 2 (2) | 0.78 (±4.32) | 0.00 (0.00-0.00) |
| **IL-9** | 1 (1) | NA | NA | 1 (1) | NA | NA |
| **IL-10** | 2 (2) | 0.12 (±1.10) | 0.00 (0.00-0.00) | 3 (4) | 0.14 (±1.23) | 0.00 (0.00-0.00) |
| **IL-12p70** | 0 (0) | NA | NA | 0 (0) | NA | NA |
| **IL-13** | 1 (1) | NA | NA | 3 (4) | 0.06 (±0.39) | 0.00 (0.00-0.00) |
| **IL-15** | 6 (7) | 3.74 (±15.56) | 0.00 (0.00-0.00) | 4 (5) | 1.29 (±6.41) | 0.00 (0.00-0.00) |
| **IL-17A** | 14 (17) | 2.13 (±7.77) | 0.00 (0.00-0.00) | 16 (19) | 1.20 (±6.54) | 0.00 (0.00-0.00) |
| **IL-18** | 35 (42) | 2.64 (±5.02) | 0.00 (0.00-2.95) | 55 (66) | 9.88 (±12.84) | 4.56 (0.00-14.72) |
| **IL-21** | 7 (8) | 25.35 (±132.29) | 0.00 (0.00-0.00) | 6 (7) | 20.53 (±108.79) | 0.00 (0.00-0.00) |
| **IL-22** | 5 (6) | 63.68 (±361.96) | 0.00 (0.00-0.00) | 5 (6) | 36.21 (±211.76) | 0.00 (0.00-0.00) |
| **IL-23** | 0 (0) | NA | NA | 0 (0) | NA | NA |
| **IL-27** | 5 (6) | 2.99 (±15.35) | 0.00 (0.00-0.00) | 5 (6) | 2.45 (±16.38) | 0.00 (0.00-0.00) |
| **IL-31** | 0 (0) | NA | NA | 0 (0) | NA | NA |
| **IP-10/CXCL10** | 83 (100) | 19.94 (±13.03) | 17.43 (11.66-26.31) | 83 (100) | 18.42 (±10.27) | 15.23 (11.87-21.71) |
| **MCP-1/CCL2** | 80 (96) | 29.77 (±23.70) | 23.86 (11.47-44.51) | 79 (95) | 34.26 (±27.53) | 27.02 (15.48-50.01) |
| **MIP-1α/CCL3** | 23 (28) | 3.44 (±8.74) | 0.00 (0.00-1.34) | 17 (21) | 2.84 (±8.50) | 0.00 (0.00-0.00) |
| **MIP-1β/CCL4** | 83 (100) | 67.04 (±102.81) | 42.17 (33-37-65.20) | 83 (100) | 72.88 (±93.55) | 50.82 (34.94-72.11) |
| **RANTES/CCL5** | 83 (100) | 4.39 (±2.06) | 4.03 (2.75-5.64) | 83 (100) | 4.60 (±2.00) | 4.08 (3.13-5.42) |
| **SDF-1α/CXCL12** | 83 (100) | 299.25 (±345.30) | 239.78 (200.65-285.37) | 83 (100) | 282.83 (±297.43) | 232.54 (197.30-277.98) |
| **TNF-α** | 10 (12) | 0.28 (±1.33) | 0.00 (0.00-0.00) | 9 (11) | 0.19 (±0.97) | 0.00 (0.00-0.00) |
| **TNF-β** | 0 (0) | NA | NA | 0 (0) | NA | NA |

GM-CSF = granulocyte-macrophage colony-stimulating factor, GRO = growth-regulated oncogene, IFN = interferon, IL = interleukin, IL-1RA = interleukin-1 receptor antagonist, IP = interferon γ-induced protein, IQR = interquartile range, MCP = monocyte chemoattractant protein, MIP = macrophage inflammatory protein, NA = not applicable, RANTES = regulated upon activation, normal T cell expressed and secreted, SD = standard deviation, SDF = stromal cell-derived factor, TNF = tumor necrosis factor.

**Supplementary Table 5.** Comparison of detection rates of cytokines and median cytokine concentrations between patients in different stages of disease, including patients with resectable, locally advanced (LAPC), and metastatic disease.

| **Before start of FOLFIRINOX** | | | | | | | | |
| --- | --- | --- | --- | --- | --- | --- | --- | --- |
|  | **Resectable disease, *n*=28** | | **LAPC, *n*=35** | | **Metastatic disease, *n*=20** | |  | |
| **Cytokine** | **Detection rate (%)** | **Median concentration, pg/mL (IQR)** | **Detection rate (%)** | **Median concentration, pg/mL (IQR)** | **Detection rate (%)** | **Median concentration, pg/mL (IQR)** | ***P^a^*** | ***P^b^*** |
| Eotaxin | 28 (100) | 67.52 (34.46-93.07) | 35 (100) | 58.11 (46.18-104.77) | 20 (100) | 54.96 (36.26-87.78) | 1.000 | 0.646 |
| IL-1β | 28 (100) | 0.24 (0.15-2.81) | 35 (100) | 0.30 (0.21-0.44) | 20 (100) | 0.24 (0.16-0.53) | 1.000 | 0.688 |
| IL-1RA | 28 (100) | 445.35 (365.20-779.98) | 35 (100) | 687.66 (413.69 (979.88) | 20 (100) | 529.40 (324.32-779.53) | 1.000 | 0.170 |
| sIL-2R | 28 (100) | 3152.50 (2105.75-3898.50) | 35 (100) | 2749.00 (1874.00-4133.00) | 20 (100) | 3093.00 (2309.75-6417.50) | 1.000 | 0.213 |
| IL-7 | 19 (68) | 0.42 (0.00-1.09) | 26 (74) | 0.59 (0.00-1.11) | 14 (70) | 0.59 (0.00-1.46) | 0.849 | 0.768 |
| IL-18 | 28 (100) | 342.88 (276.26-444.16) | 35 (100) | 285.88 (238.49-510.41) | 20 (100) | 343.59 (255.63-442.02) | 1.000 | 0.834 |
| IP-10 | 28 (100) | 16.47 (10.46-27.34) | 35 (100) | 17.51 (12.32-27.05) | 20 (100) | 17.29 (11.07-22.39) | 1.000 | 0.763 |
| MCP-1 | 28 (100) | 24.72 (14.83-45.64) | 33 (94) | 24.43 (10.47-47.90) | 19 (95) | 17.91 (9.54-36.19) | 0.449 | 0.316 |
| MIP-1β | 28 (100) | 38.28 (29.79-66.75) | 35 (100) | 42.51 (34.09-59.99) | 20 (100) | 49.83 (34.23-74.73) | 1.000 | 0.365 |
| RANTES | 28 (100) | 3.90 (2.66-5.24) | 35 (100) | 3.91 (2.86-6.10) | 20 (100) | 4.37 (2.61-6.14) | 1.000 | 0.536 |
| SDF-1α | 28 (100) | 228.20 (166.02-276.13) | 35 (100) | 241.70 (218.50-301.43) | 20 (100) | 250.58 (203.64-320.45) | 1.000 | 0.234 |
| **After 1 cycle of FOLFIRINOX** | | | | | | | | |
|  | **Resectable disease, *n*=28** | | **LAPC, *n*=35** | | **Metastatic disease, *n*=20** | |  | |
| **Cytokine** | **Detection rate (%)** | **Median concentration, pg/mL (IQR)** | **Detection rate (%)** | **Median concentration, pg/mL (IQR)** | **Detection rate (%)** | **Median concentration, pg/mL (IQR)** | ***P^a^*** | ***P^b^*** |
| Eotaxin | 28 (100) | 69.13 (36.95-88.81) | 35 (100) | 72.12 (43.98-113.45) | 20 (100) | 73.05 (36.78-84.09) | 1.000 | 0.772 |
| IL-1β | 28 (100) | 0.36 (0.21-0.48) | 35 (100) | 0.42 (0.30-0.82) | 20 (100) | 0.38 (0.30-0.45) | 1.000 | 0.301 |
| IL-1RA | 28 (100) | 645.10 (319.59-3019.30) | 35 (100) | 1906.93 (1620.91-2901.32) | 20 (100) | 1486.39 (980.62-2967.40) | 1.000 | 0.093 |
| sIL-2R | 28 (100) | 4132.00 (3263.75-5244.50) | 35 (100) | 4313.00 (3204.00-7452.00) | 20 (100) | 5473.00 (3247.00-8393.50) | 1.000 | 0.254 |
| IL-7 | 20 (71) | 0.35 (0.00-1.07) | 24 (69) | 0.42 (0.00-0.93) | 14 (70) | 0.59 (0.00-1.22) | 0.970 | 0.655 |
| IL-18 | 28 (100) | 507.22 (409.16-806.23) | 35 (100) | 547.92 (430.95-984.96) | 20 (100) | 629.29 (458.19-980.39) | 1.000 | 0.319 |
| IP-10 | 28 (100) | 15.73 (11.89-22.78) | 35 (100) | 15.67 (11.80-20.72) | 20 (100) | 14.16 (11.83-23.04) | 1.000 | 0.947 |
| MCP-1 | 28 (100) | 26.94 (18.73-47.70) | 32 (91) | 32.98 (15.48-55.35) | 19 (95) | 23.36 (13.98-37.88) | 0.287 | 0.518 |
| MIP-1β | 28 (100) | 42.57 (29.12-60.18) | 35 (100) | 57.64 (38.11-78.78) | 20 (100) | 59.74 (47.31-81.23) | 1.000 | 0.066 |
| RANTES | 28 (100) | 3.89 (3.10-5.36) | 35 (100) | 4.20 (3.14-5.40) | 20 (100) | 4.62 (3.07-5.75) | 1.000 | 0.796 |
| SDF-1α | 28 (100) | 211.94 (190.08-272.12) | 35 (100) | 242.84 (197.30-280.45) | 20 (100) | 237.41 (204.28-281.22) | 1.000 | 0.535 |

IL = interleukin, IL-1RA = interleukin-1 receptor antagonist, IP-10 = interferon gamma-induced protein 10, MCP = monocyte chemoattractant protein, MIP = macrophage inflammatory protein. ^a^ *P*-value for the comparison of detection rates, calculated by Chi squared tests. ^b^ *P*-value for the comparison cytokine concentrations, calculated by Kruskal-Wallis tests.

**Supplementary Table 6.** Univariable and multivariable Cox proportional hazards model for overall survival (OS) after FOLFIRINOX.

|  | **Univariable** | | **Multivariable** | |
| --- | --- | --- | --- | --- |
| **Variable** | **HR for OS**  **(95% CI)** | ***P*** | **HR for OS**  **(95% CI)** | ***P*** |
| **CA19-9 at baseline**  **(per 100 kU/L)** | 1.00 (1.00-1.01) | 0.021^a^ | 1.00 (1.00-1.01) | 0.044^a^ |
| **RECIST response outcome**  **Disease control**  **Progressive disease** | Ref  7.37 (3.84-14.15) | <0.001^a^ | Ref  6.66 (2.96-14.98) | <0.001^a^ |
| **IL-1RA after 1 cycle of FOLFIRINOX**  **<median**  **>median** | Ref  0.64 (0.38-1.09) | 0.098 | Ref  0.93 (0.47-1.85) | 0.834 |
| **sIL-2R before start of FOLFIRINOX**  **<median**  **>median** | Ref  1.55 (0.93-2.59) | 0.093 | Ref  1.07 (0.59-1.93) | 0.823 |
| **IL-7 after 1 cycle of FOLFIRINOX**  **<median**  **>median** | Ref  1.57 (0.93-2.65) | 0.090 | Ref  2.14 (1.20-3.80) | 0.010^a^ |
| **IL-18 before start of FOLFIRINOX**  **<median**  **>median** | Ref  1.56 (0.93-2.60) | 0.090 | Ref  1.12 (0.62-2.02) | 0.714 |
| **IL-18 after 1 cycle of FOLFIRINOX**  **<median**  **>median** | Ref  1.57 (0.94-2.62) | 0.084 | Ref  2.00 (1.11-3.60) | 0.020^a^ |
| **MIP-1β after 1 cycle of FOLFIRINOX**  **<median**  **>median** | Ref  0.63 (0.38-1.05) | 0.076 | Ref  0.51 (0.28-0.92) | 0.025^a^ |

CA19-9 = carbohydrate antigen 19-9, CI = confidence interval, HR = hazard ratio, IL = interleukin, IL-1RA = interleukin-1 receptor antagonist, MIP = macrophage inflammatory protein, Ref = reference, sIL2R = soluble interleukin-2 receptor. ^a^ Significant *P*-value.

**
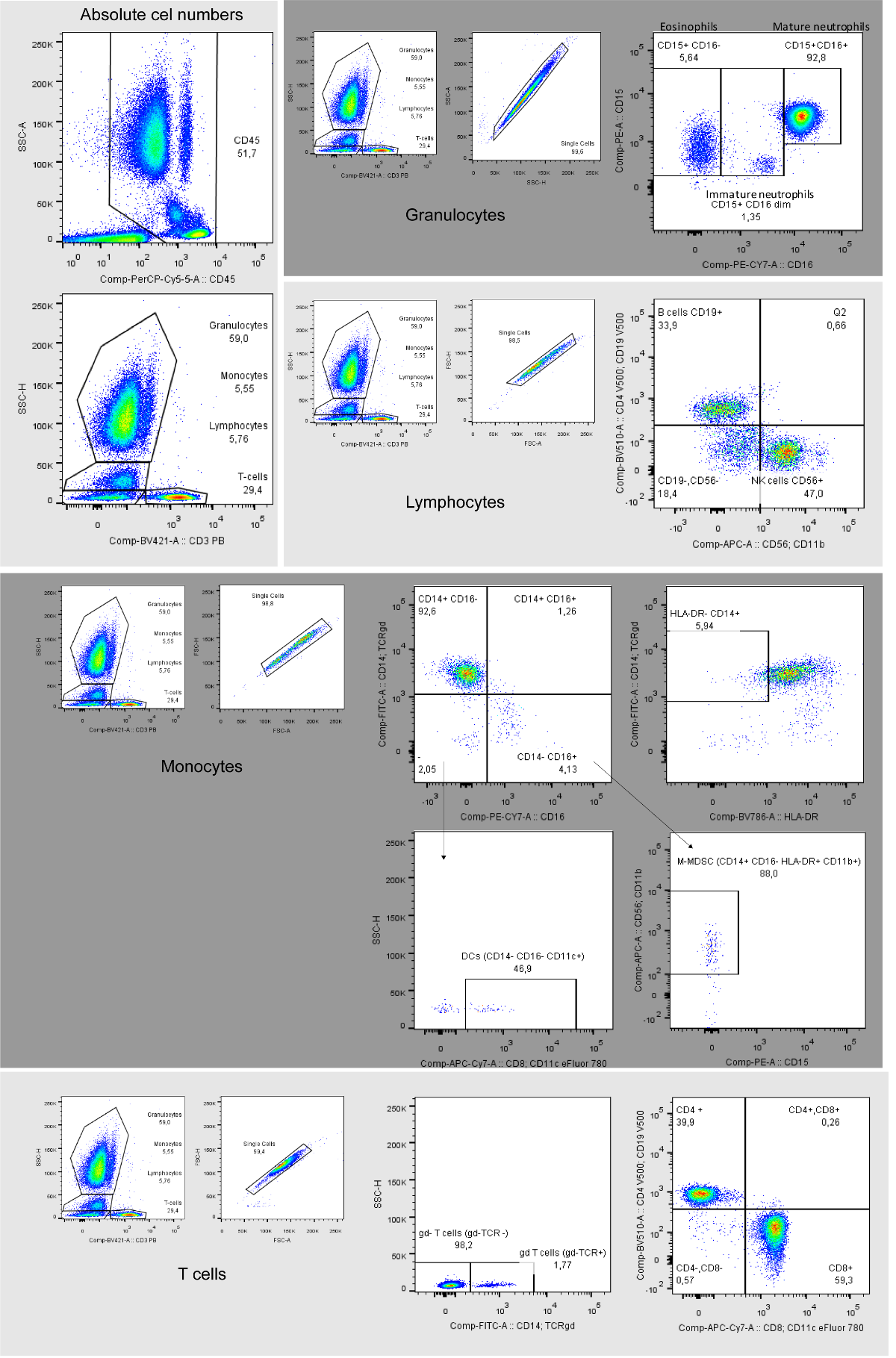
Supplementary Figure 1.** Flow cytometry gating strategy for absolute immune cell numbers and immune cell subsets used with FlowJo software.

**Supplementary Figure 2.** Differences in circulating cytokine concentrations between patients with disease control and patients with progressive disease during FOLFIRINOX treatment.


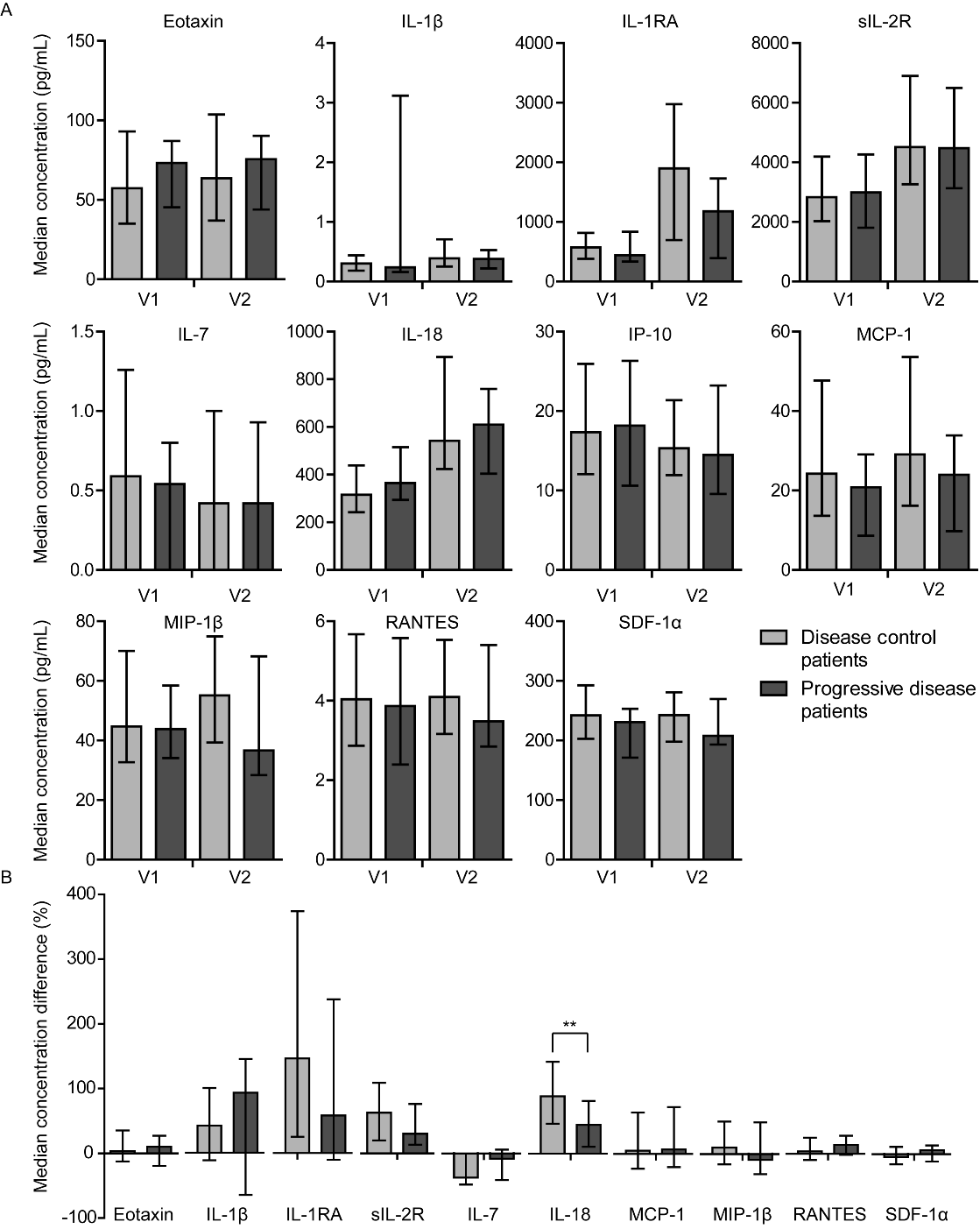


(A) Concentrations of serum cytokines in patients with disease control (*n*=64) and patients with progressive disease (*n*=19) before start of FOLFIRINOX (V1) and after one cycle of FOLFIRINOX (V2). (B) Percentage increase of serum cytokine concentrations after one cycle of FOLFIRINOX. Treatment-induced IL-18 increase was higher in patients with disease control compared to patients with progressive disease after FOLFIRINOX. ** = *P*<0.01, calculated with Mann-Whitney U test. Data is presented as median cytokine concentrations or cytokine concentration differences with error bars representing the interquartile range.


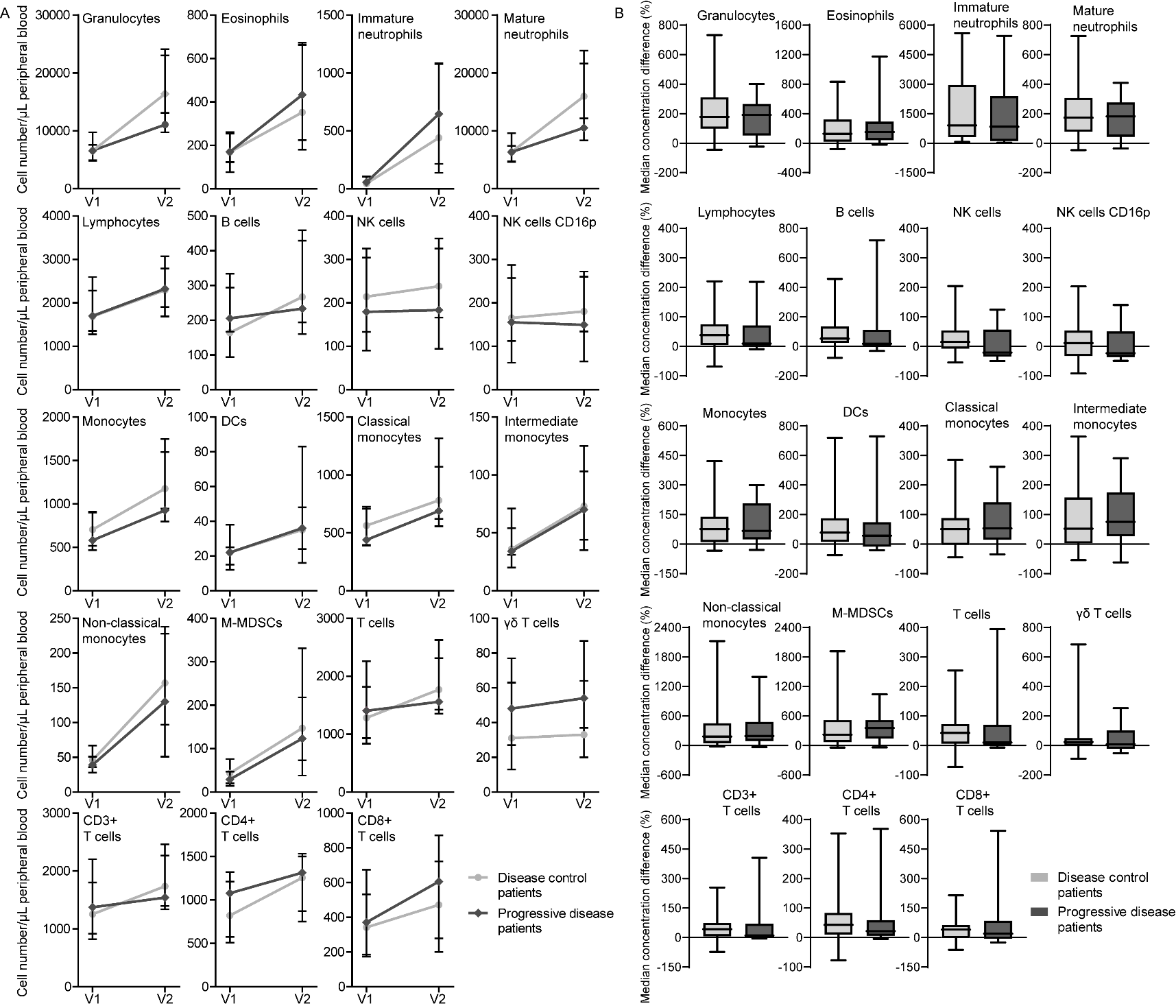
**Supplementary Figure 3.** Differences in circulating immune cell numbers in peripheral blood between patients with disease control and patients with progressive disease during FOLFIRINOX treatment.

(A) Circulating immune cell numbers in patients with disease control and patients with progressive disease before start of FOLFIRINOX (V1) and after one cycle of FOLFIRINOX (V2). (B) Percentage increase of immune cell numbers after one cycle of FOLFIRINOX in patients with disease control and patients with progressive disease.

**Supplementary Figure 4.** Kaplan-Meier curves for overall survival for patients with cytokine levels below and above the median cytokine concentration measured after one cycle of FOLFIRINOX.


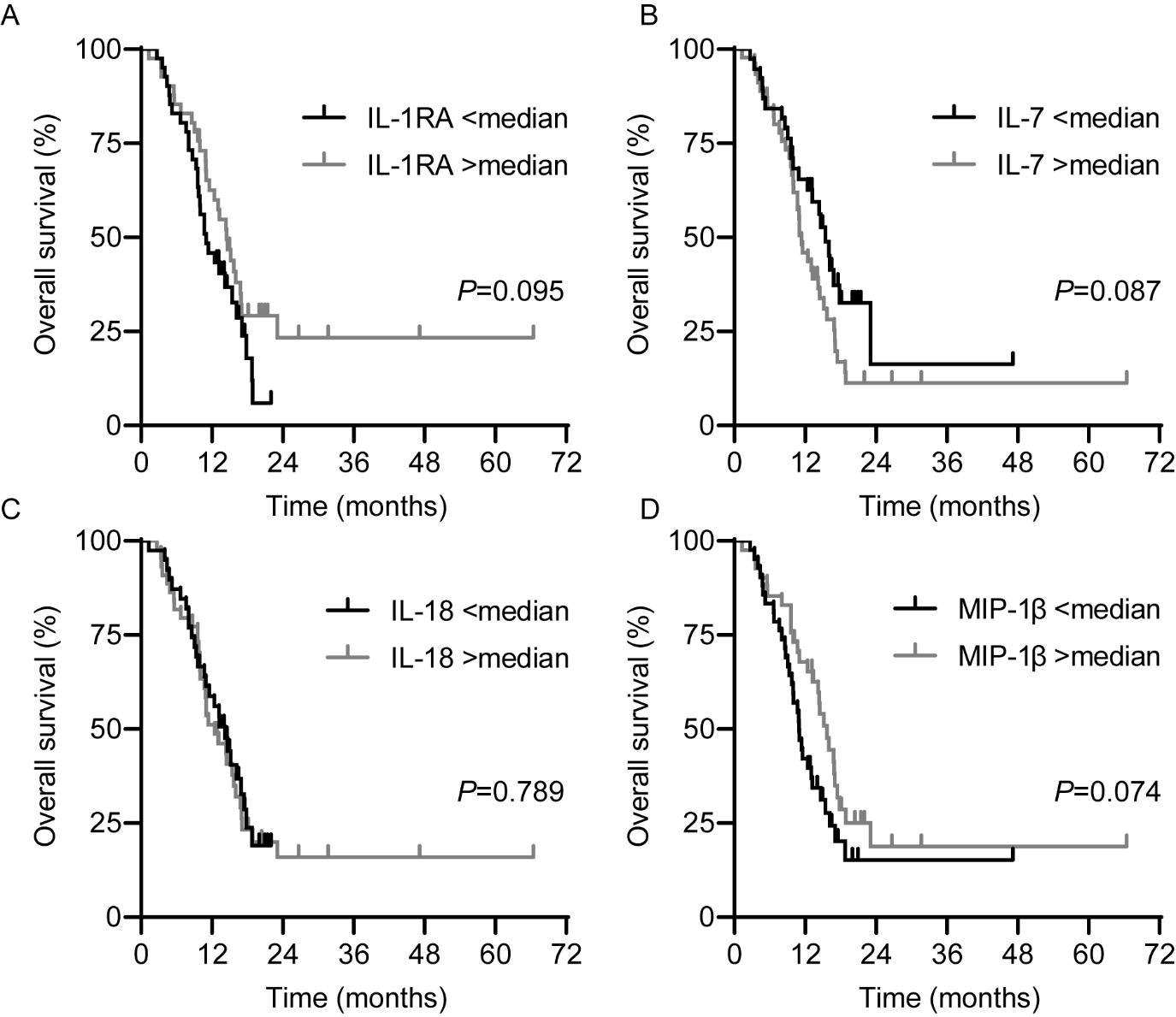


(A) Kaplan-Meier curves for interleukin-1 receptor antagonist (IL-1RA), (B) interleukin-7, (C) interleukin-18, and (D) MIP-1β.

High IL-1RA and MIP-1β and low IL-7 concentrations after one cycle of FOLFIRINOX show a trend of better overall survival after FOLFIRINOX treatment, though borderline significant. IL-18 levels after FOLFIRINOX do not influence overall survival.
